# Modulation of VEGF and IL-2 in radiation-induced brain injury: neuroprotective effects of melatonin and ascorbic acid in rats

**DOI:** 10.1007/s11060-026-05484-9

**Published:** 2026-02-27

**Authors:** Gulhan Guler Avci, Fikret Gevrek, Sefa Colak, Asiye Yanci

**Affiliations:** 1 Department of Radiation Oncology, Tokat Gaziosmanpaşa University Faculty of Medicine, Tokat, Türkiye; 2 Department of Histology and Embryology, Tokat Gaziosmanpaşa University Faculty of Medicine, Tokat, Türkiye; 3 Department of Oral, Dental and Maxillofacial Surgery, Tokat Gaziosmanpaşa University Faculty of Dentistry, Tokat, Türkiye

**Keywords:** Radiation-induced brain injury, Melatonin, Ascorbic acid, Vascular damage, VEGF, IL-2

## Abstract

**Purpose:**

This study investigates the neuroprotective effects of melatonin and ascorbic acid, alone and in combination, on radiation-induced brain injury, specifically focusing on vascular and immunological modulations.

**Methods:**

Thirty-three male Wistar-Albino rats were assigned to five groups: Control (G1), Radiotherapy (RT) only (single dose 20 Gy) (G2), RT + Melatonin (20 mg/kg) (G3), RT + vitamin C (100 mg/kg) (G4), and RT + Melatonin + Vitamin C (G5). The treatments were administered intraperitoneally the day after RT. Brain tissues were evaluated 28 days post-irradiation for histopathological neural damage, vasocongestion, and immunohistochemical expression of vascular endothelial growth factor (VEGF) and interleukin-2 (IL-2).

**Results:**

High-dose RT significantly increased vasocongestion and neuronal damage while suppressing VEGF expression and elevating IL-2 levels (*p* < 0.001). Both melatonin and ascorbic acid effectively reduced histopathological injury, maintained VEGF levels, and balanced IL-2 expression compared to the RT-only group (*p* < 0.05). However, combining both agents provided no significant synergistic advantage over monotherapies. Notably, single-fraction high-dose RT resulted in a marked suppression of VEGF expression, suggesting an impaired vascular synthetic capacity rather than a compensatory angiogenic response.

**Conclusion:**

Melatonin and ascorbic acid provide substantial neuroprotection against radiation-induced brain injury by preserving vascular integrity and modulating local immune responses. These antioxidants represent potential therapeutic strategies for minimizing RT-induced neurotoxicity.

## Introduction

Radiotherapy (RT), a standard adjuvant therapy for primary and metastatic brain tumors, is also known to induce cytotoxic effects on healthy brain tissues. It can lead to the development of acute, subacute, and late-stage neurological damage [1]. For years, researchers have been searching for strategies to reduce the early and late effects of RT. Melatonin and ascorbic acid have attracted attention as potential neuroprotective agents against radiation damage due to their potent antioxidant and anti-inflammatory properties. Melatonin is a molecule synthesized endogenously in the human body and has the potential to reduce the toxic effects associated with chemotherapy and RT [2, 3]. In addition to its fundamental role in regulating the circadian rhythm, melatonin has been shown to modulate endothelial damage and changes in the immune response that develop after radiation [4–6]. Melatonin has been reported to protect against neuronal apoptosis by preserving mitochondrial function and scavenging free radicals [7]. Ascorbic acid plays an important role in suppressing oxidative stress and preserving vascular endothelial integrity [8].

However, most existing studies have focused on the effects of melatonin alone. Data examining the potential synergistic effects of combining melatonin and ascorbic acid to prevent vascular damage after high-dose RT and its implications for immune-vascular markers, such as vascular endothelial growth factor (VEGF) and interleukin-2 (IL-2), are limited. Since radiation damage to the brain most commonly occurs through vascular structures, studying the relationship between vascular congestion, neuronal damage, and VEGF levels would address an important gap in the literature. While fractionated or low-dose RT has been associated with compensatory VEGF upregulation, the effects of high-dose single-fraction RT on VEGF synthesis capacity in brain tissue remain unclear [2–7]. One of the most common complications observed in brain parenchyma, particularly after high-dose RT, is damage to the vascular system. This condition is characterized by endothelial cell swelling, vascular dilation, congestion, and impaired blood-brain barrier permeability. Vascular endothelial growth factor plays a critical role in pathological angiogenesis and vascular permeability processes. It is also a key marker for monitoring the destructive effects of radiation on the vascular network [9, 10]. Conversely, IL-2 is an important cytokine involved in regulating the immune response and indicative of T cell proliferation. IL-2 is considered a marker of microglial activation and of the local immune response that develops in response to tissue damage induced by RT [11].

This study aims to evaluate the effects of melatonin and ascorbic acid, both alone and in combination, on histopathological damage, the vascular response, and the immune response in a rat model of high dose radiation-induced brain damage. Congestion and neuron damage, as well as VEGF and IL-2 expression levels, were examined in this context. We hypothesized that high-dose single-fraction RT suppresses VEGF synthesis capacity through direct endothelial injury, and that treatment with melatonin and ascorbic acid mitigates radiation-induced brain injury by preserving vascular and immunological homeostasis rather than promoting angiogenesis.

## Materials and methods

### Experimental animals

This study included a total of 33 young adult male Wistar-Albino rats, weighing between 350 and 400 g. The animals were obtained from the Experimental Medical Research Unit of Gaziosmanpaşa University and were kept under standardized laboratory conditions throughout the study. The ambient temperature was set between 20 and 22 °C, and the relative humidity was maintained between 40% and 60%. A 12-hour light–dark cycle was used to replicate normal circadian rhythms. Five rats were assigned to the control group, while the remaining 28 were assigned to the experimental groups. All experimental groups underwent head irradiation (a single dose of 20 Gy) [12].

Group 1 (control, *n* = 5) received no treatment.

Group 2 (G2; RT, *n* = 7) received a single 20 Gy dose of RT.

Group 3 (G3; RT + MEL; *n* = 7) received the same RT protocol as Group 2, followed by melatonin administration at a dose of 20 mg/kg/day for 27 consecutive days beginning the day after RT.

Group 4 (G4; RT + Cvit; *n* = 7) received the same RT protocol as Group 2, followed by ascorbic acid administration at a dose of 100 mg/kg/day for 27 consecutive days beginning the day after RT.

Group 5 (G5; RT + MEL + Cvit; *n* = 7) received the same RT protocol as Group 2, followed by the combined administration of melatonin (20 mg/kg/day) and ascorbic acid (100 mg/kg/day) for 27 consecutive days, beginning the day after RT.

All animals underwent the same anesthesia protocol and follow-up period.

### Irradiation of animals

**Fig. 1:**
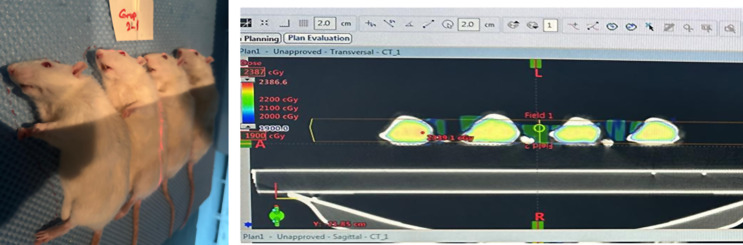
The radiation and planning dose distributions for animals (95% isodose volume, Dmax: 23.8 Gy)

Radiotherapy was administered at the Department of Radiation Oncology, Gaziosmanpaşa University, Tokat, using a Varian Clinac DHX 5776 linear accelerator (Varian Medical Systems, Palo Alto, CA, USA) under the supervision of a radiation oncologist and a medical physicist. Prior to irradiation, animals were anesthetized with intraperitoneal ketamine hydrochloride (100 mg/kg) and xylazine hydrochloride (10 mg/kg) to ensure adequate immobility. Rats were placed in a lateral recumbent position on a foam support platform mounted on the treatment bed. Simulation was performed by acquiring planning computed tomography of four animals in a side-by-side arrangement simultaneously (Fig. 1). To maintain a fixed position during irradiation, the outer contours of each animal were marked on the foam surface with a pen. Special care was taken to ensure accurate positioning of the cranial region within the designated irradiation field.

Irradiation was administered using 6 MV photon beams from two opposing anterior-posterior (AP-PA) fields using a 3D conformal technique. The treatment field was collimated using appropriate multi-leaf collimator (MLC) shaping so that only the head region remained within the radiation field, while the rest of the body was excluded from direct radiation exposure. A single fraction dose of 20 Gy was prescribed to the brain target volume. A schematic representation of the irradiation setup is shown in Fig. 1.

### Melatonin and ascorbic acid administration

Melatonin (Sigma-Aldrich, St. Louis, Missouri, USA) was administered to rats in G3 and G5 via intraperitoneal injection at a dose of 20 mg/kg/day for 27 days [13]. Ascorbic acid (Redox-C 500 mg/5 mL; Mefar İlaç San. ve Tic. Şti., Istanbul, Türkiye) was administered intraperitoneally at a dose of 100 mg/kg daily for 27 days to rats in G4 and G5 [14]. The intraperitoneal route was chosen to increase systemic bioavailability and ensure standardized administration. Melatonin and ascorbic acid were administered approximately two hours after the start of the light phase (around 10:00 a.m.), according to the rats’ 12-hour light/dark cycle. Twenty-eight days after RT, all animals were euthanized with high-dose intraperitoneal injections of 100 mg/kg of ketamine HCl and 10 mg/kg of xylazine. Brain tissue was removed from all rats.

### Ethical approval

This research has been endorsed by the Local Ethics Committee for Animal Experiments at Tokat Gaziosmanpaşa University (approval number 51879863-38, dated June 12, 2024) and registered as a preclinical trial (number 2024 HAYDEK-11). All procedures were executed in accordance with the institutional guidelines of the Experimental Medical Research Centre and the university’s regulations on animal experimentation.

### Histological procedures

#### Tissue processing

At the end of the experiment, the rats’ brains were removed and fixed in a 4% buffered neutral formalin solution. They were then subjected to routine histological tissue processing. The left hemispheres of all rats were embedded in paraffin blocks in the same orientation. Thin sequential Sect. (4 μm thick) were cut from the paraffin-embedded brains using a rotary microtome (Leica RM2135, Germany) and mounted on slides. For histopathological analysis, the sections were stained with hematoxylin and eosin. For immunohistochemical analysis, the sections were stained according to the indirect immunohistochemistry staining protocol. This prepared the slides for microscopic analysis.

#### Histopathological analyses

Research was conducted on hematoxylin and eosin-stained tissue sections using a Nikon Eclipse E600 light microscope (Tokyo, Japan) with NIS-Elements computer-assisted software (HASP ID: 6648AA61). Tissue sections were evaluated for vasocongestion and neuronal damage in the brain tissue cortex under a light microscope. Analyses were performed by counting cells systematically in five different areas of the cortex, based on the staining intensity of neurons, in an average of five to six consecutive sections from each individual. Cell counts were performed on 40x objective images of each area. The criteria used to grade neuronal staining are listed in Table 1. Additionally, tissue sections were scored semiquantitatively from 0 to 3 for vasocongestion according to the criteria in Table 2. The analyses were performed as a blinded study using a coding system. Weighted mean values were obtained for each individual from the counts, and group mean values were calculated and statistically compared.

**Table 1:** Criteria used in the assessment of neuronal damage [15, 16]

Scores	Staining criterion feature
1	Normal staining
2	Moderately intense staining
3	Intense (dark) staining

**Table 2:** Vasocongestion scoring criteria [17, 18]

Scores	Criteria
Grade 0	Normal
Grade 1	Mild
Grade 2	Moderate
Grade 3	Severe

#### Hematoxylin-eosin staining

In summary, the steps for hematoxylin-eosin staining of thin tissue sections taken from formalin-fixed, paraffin-embedded brain tissues are as follows: The steps are: deparaffinization, rehydration, soaking in hematoxylin solution, washing in running water, immersion in acid alcohol and distilled water, soaking in eosin dye solution, washing in distilled water, passing through an alcohol series (80%, 90%, 95%, and 99%), and passing through xylenes. Finally, the slides are sealed with a coverslip using Entellan.

#### Immunohistochemical analysis

Immunohistochemical staining intensities were determined using a Nikon Eclipse 200 research light microscope at 40x magnification and the NIS-Element program, based on the scoring criteria in Table 2. Unstained and stained cells in sections stained for VEGF and IL-2 were counted according to the intensity of the staining reaction, using the following grading criteria: score 0 = negative staining, score 1 + = weak staining, score 2 + = moderate staining, and score 3 + = strong staining [19]. The mean group results were converted to H-score values using the formula [∑Pi(i + l)]. In the formula, i represents the staining intensity score, and pi represents the percentage of stained cells. An experienced histologist who was blinded to the groups performed the analyses.

### Immunohistochemical staining

Thin tissue sections obtained from formalin-fixed, paraffin-embedded brains were stained using an indirect immunohistochemistry protocol to detect immune expression of the VEGF and IL-2 molecules. The process was carried out as follows: After deparaffinization in a 60 °C oven, the sections were rehydrated in a series of alcohols (100%, 90%, 80%, and 70%), followed by distilled water. After antigen retrieval in a microwave with 10 mM citric acid, endogenous peroxidase was blocked with a 3% hydrogen peroxide solution for 10 min. After washing in phosphate-buffered saline (PBS) three times for five minutes, the perimeter was outlined with a hydrophobic pen, and non-immunological blocking serum was applied. The slides were then incubated in a humid, dark environment for 15 min. VEGF and IL-2 primary antibodies (1:100 bs-0279R BIOSS, USA; 1:150 E-AB-40275 Elabscience, USA) were added to the sections, which were then incubated overnight at approximately 4 °C in a closed, humid environment. After washing with PBS (three times for five minutes each), a biotinylated secondary antibody was added and incubated for 45 min at room temperature in a closed, humid, dark environment. After washing with PBS (three times for five minutes each), a second secondary antibody (streptavidin-horseradish peroxidase conjugated reagent [HRP]) was added and incubated in a closed, humid environment for 30 min. After washing with PBS (three times for five minutes each), the sections were stained with an aminoethylcarbazole (AEC) chromogen solution. Then, a counterstain with hematoxylin was performed. After passing through distilled water, the slide was sealed with a water-based sealing solution (Aquose Mount Reagent). For the negative control section, PBS was used instead of the primary antibody, and no staining occurred.

### Statistical analysis

The data obtained from the study were analyzed using the Statistical Package for the Social Sciences (SPSS) for Windows, version 15. The Kruskal-Wallis test was used to detect differences between groups. When the test revealed a significant difference, the Mann-Whitney U test was applied to determine which groups differed. The data are expressed as the mean ± standard deviation (SD), and are considered statistically significant when the probability value is *p* < 0.05.

## Results

### Histopathological findings

#### Vasocongestion

**Fig. 2:**
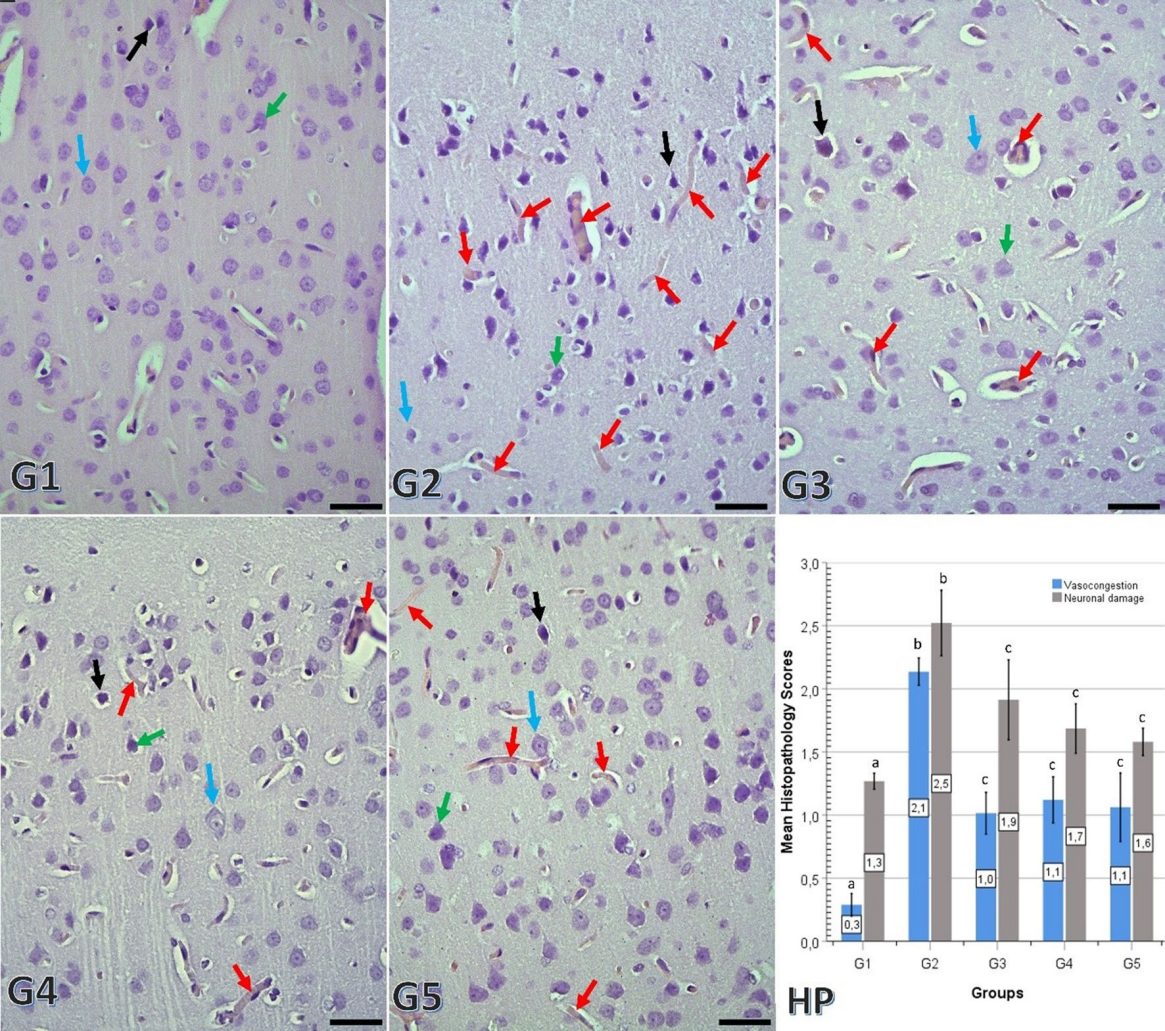
Representative microscopic images showing neuronal damage and vasocongestion in the experimental groups (G1, G2, G3, G4, and G5), as well as a graphical comparative view of the mean group values of these histological damage scores (HP). The black arrow indicates a severely damaged neuron that is heavily stained; the green arrow indicates a moderately damaged neuron; and the magenta arrow indicates a normal neuron that is lightly stained. Red arrows indicate vasocongestion. Bars represent mean ± SD values. Different lowercase letters (a, b, c) above the bars indicate statistically significant differences between groups (*p* < 0.05, Kruskal–Wallis test followed by Mann–Whitney U post hoc analysis), whereas identical letters indicate no statistically significant difference between groups. (Hematoxylin and eosin staining; scale bars: 25 μm)

Significant differences in vasocongestion were observed among the groups (Fig. 2). The control group (G1) demonstrated significantly lower vasocongestion compared to all other groups (*p* < 0.001 for all comparisons). The radiation-only group (G2) showed the highest vasocongestion scores and had significantly higher levels than G3, G4, and G5 (*p* < 0.001 for all comparisons). No statistically significant differences were detected among G3, G4, and G5 (G3–G4: *p* = 0.283; G3–G5: *p* = 0.629; G4–G5: *p* = 0.549).

#### Neuronal damage

Multiple comparisons between groups in terms of neuronal damage revealed statistically significant differences (Fig. 2). The G1 group had normal-appearing neural tissue, with no significant neuronal damage observed (G1–G2: *p* < 0.001; G1–G3: *p* = 0.014; G1–G4: *p* = 0.009; G1–G5: *p* = 0.001). The G2 group had the highest neuronal damage scores and showed significantly more damage than the G1, G3, G4, and G5 groups (*p* ≤ 0.021 for all comparisons). These results indicated that the G2 group experienced significant neuronal degeneration. No statistically significant differences were detected among G3, G4, and G5 (G3–G4: *p* = 0.773; G3–G5: *p* = 0.280; G4–G5: *p* = 0.938).

### Immunohistochemical findings

#### VEGF immune expression

**Fig. 3:**
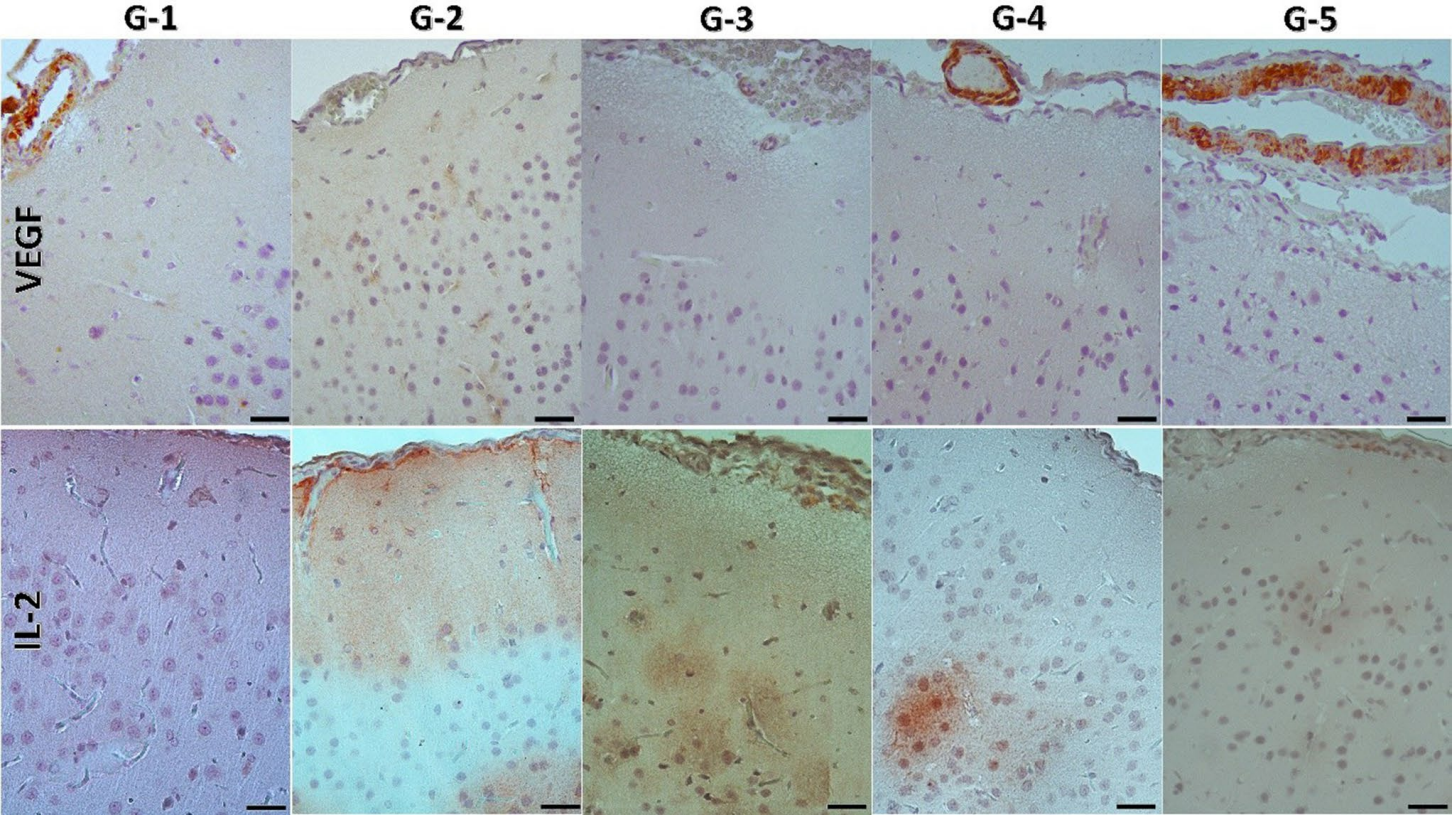
Representative microscopic images (IHC-AEC; Scale Bars: 25 μm) of the immunological expression of VEGF (Top Row) and IL-2 (Bottom Row) molecules from the working groups (G1, G2, G3, G4, and G5)

**Fig. 4:**
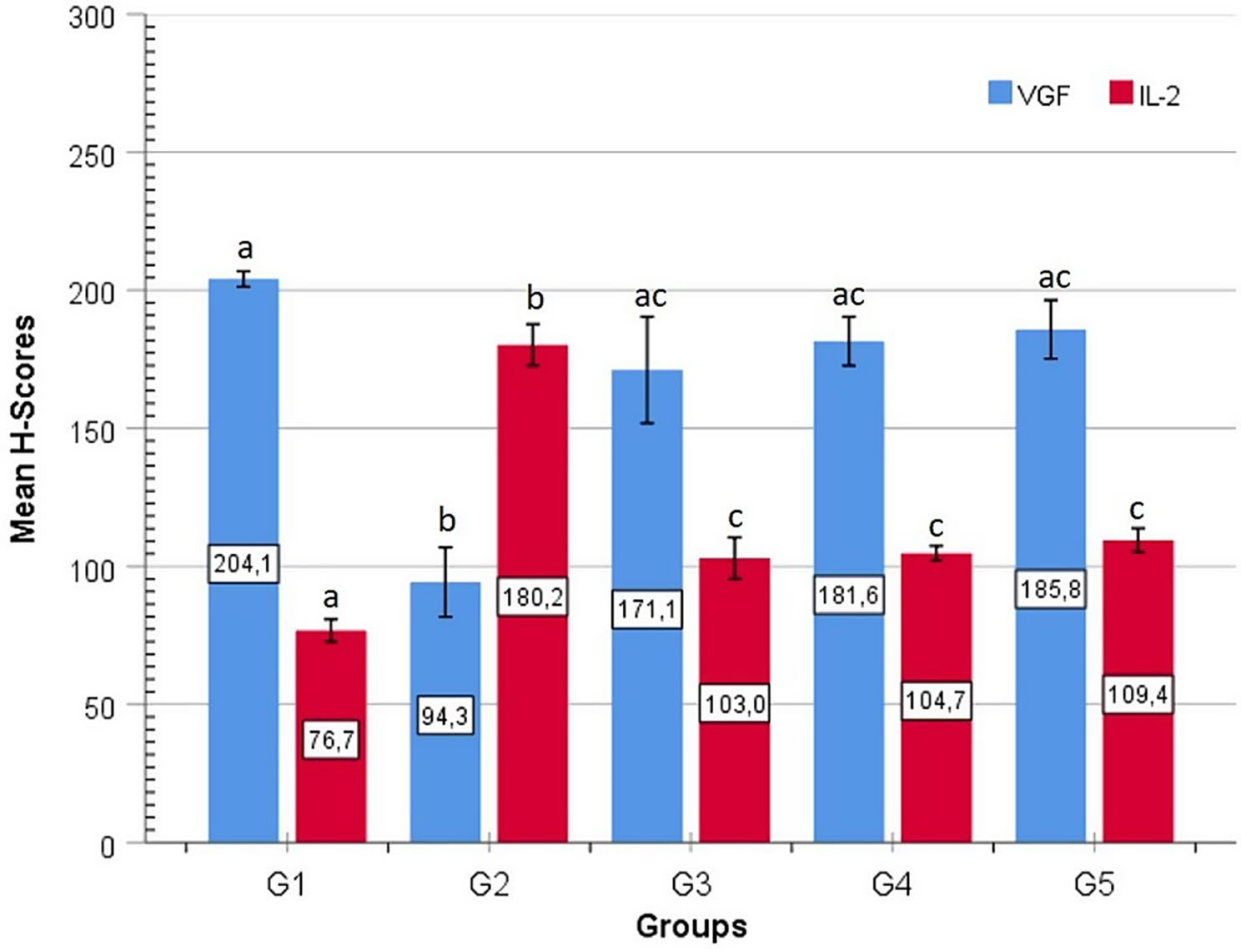
Graphical comparative view of the immunological expression of VEGF (VGF, blue bar) and IL-2 (red bar) based on group average H-scores. Identical letters on the bars indicate statistical similarity for each parameter, and different letters indicate differences

Significant differences in VEGF immunohistochemical expression were observed between groups (Figs. 3 and 4). The G2 group showed significantly lower VEGF expression than all the other groups. The G2 group had significantly lower VEGF H-score values than the other groups (G2–G1: *p* < 0.001; G2–G3: *p* < 0.001; G2–G4: *p* < 0.001; G2–G5: *p* < 0.001). The G1 group had the highest VEGF expression values, showing significantly increased immunoreactivity compared to the G2 group (*p* < 0.001). However, no statistically significant difference in VEGF expression was found between the G1 and G3, G4, or G5 groups (*p* > 0.05). Similarly, no significant difference in VEGF immunoreactivity was observed when the G3, G4, and G5 groups were compared with each other (G3–G4: *p* = 0.549; G3–G5: *p* = 0.401; G4–G5: *p* = 0.807).

#### IL-2 immune expression

Statistically significant differences in IL-2 expression were observed among the groups (Figs. 3 and 4). The G2 group demonstrated significantly higher IL-2 H-score values compared to G1, G3, G4, and G5 (*p* < 0.001 for all comparisons). The G1 group showed significantly lower IL-2 expression than G3, G4, and G5 (G1–G3: *p* = 0.003; G1–G4: *p* = 0.002; G1–G5: *p* < 0.001). No statistically significant differences were detected between the G3, G4, and G5 groups (G3–G4: *p* = 0.825; G3–G5: *p* = 0.419; G4–G5: *p* = 0.555).

## Discussion

This study evaluated the histopathological, vascular, and immunological effects of melatonin and ascorbic acid, both alone and in combination, on RT-induced brain damage in a rat model. Findings revealed that RT alone caused marked vasocongestion and neuronal damage accompanied by a vascular inflammatory response, characterized by suppressed VEGF expression and increased IL-2 expression. In contrast, histopathological damage and the immune response were significantly reduced in groups treated with melatonin (G3), ascorbic acid (G4), or a combination of melatonin and ascorbic acid (G5).

In the current study, the selection of a single 20 Gy dose was based on its translational relevance to modern stereotactic radiosurgery and stereotactic RT techniques, where high-dose radiation is delivered in a single or limited number of fractions. While these approaches enhance tumor control, they also raise concerns regarding radiation-induced normal brain toxicity. Therefore, modeling a single high-dose exposure allows us to investigation of vascular and inflammatory mechanisms underlying neurotoxicity in contemporary RT settings. This design enhances the clinical relevance of the experimental model.

Oxidative stress, endothelial dysfunction, and local inflammation are key determinants in the pathogenesis of radiation-induced brain damage. Due to its high oxygen consumption and limited antioxidant capacity, brain tissue is susceptible to the destructive effects of radiation-induced reactive oxygen species [20]. The significant vasocongestion and increased neuronal damage observed in the G2 group in our study suggest a direct toxic effect of radiation on the vascular endothelium. This vascular damage disrupts the integrity of the blood-brain barrier and leads to secondary neuronal degeneration.

Ascorbic acid is a well-established antioxidant with documented radioprotective properties, primarily mediated through its capacity to scavenge reactive oxygen species and stabilize endothelial integrity. Experimental evidence supports its potential to mitigate radiation-induced normal tissue injury without compromising antitumor efficacy. In a recent preclinical study, Ito et al. demonstrated that ascorbic acid-2 glucoside (AA2G) attenuated radiation-induced intestinal damage in a rat bladder tumor model undergoing fractionated pelvic RT, without reducing antitumor activity [21]. However, clinical translation remains complex. In the first-in-human Phase I trial evaluating pharmacologic ascorbate administered parenterally in combination with RT and temozolomide for newly diagnosed glioblastoma, Allen et al. reported only moderate outcomes without significant improvement in the adverse effect profile [22]. This contrast between promising preclinical data and limited clinical impact suggests that the radioprotective efficacy of ascorbate may depend on dose, formulation, tumor type, and treatment context. Therefore, further mechanistic and translational studies are required to clarify its role as an adjuvant agent in radiation-induced brain injury.

Our study demonstrated a significant reduction in histopathological damage observed in both the melatonin and ascorbic acid-treated groups, revealing the potent radioprotective efficacy of these agents. Melatonin is known to suppress neuronal apoptosis by protecting mitochondrial function and scavenging free radicals, while ascorbic acid supports vascular integrity by reducing endothelial permeability [2, 8, 23]. In our study, the similar levels of protective effect observed with both agents alone can be explained by the overlap of these biological mechanisms.

One important finding of the current study is that high-dose single-fraction RT suppresses VEGF synthesis capacity in brain tissue rather than inducing a compensatory angiogenic response. This suppression likely reflects direct endothelial cell loss and impaired transcriptional activity following severe radiation-induced injury. This contrasts with studies using fractionated or low-dose RT, where VEGF upregulation is commonly observed as part of a hypoxia-driven adaptive response [24, 25]. Radiotherapy creates chronic hypoxia in brain tissue, which continuously upregulates VEGF. Hypoxia stabilizes the HIF-1α (hypoxia-inducible factor) protein. HIF-1α directly increases VEGF gene expression. The most significant effect of VEGF is increasing vascular permeability. This is the primary cause of the brain edema observed after RT [25]. Today, the reason for using bevacizumab (an anti-VEGF antibody) in the treatment of radiation-induced brain damage (radionecrosis) is as follows: Suppressing VEGF levels stops vascular leakage and rapidly reduces edema, thereby improving the patient’s condition. In our study, it is possible that the single fraction and high-dose RT suppressed VEGF synthesis capacity through endothelial cell damage and cellular loss. These findings are consistent with those of other experimental studies that have reported the suppressive effect of high-dose RT on VEGF expression. For instance, a study involving a single dose of 22 Gy of gamma irradiation applied to rat skin reported that VEGF gene expression was significantly lower in the radiation-only group than in the control group. However, VEGF expression increased significantly with the application of hesperidin, an antioxidant [26]. These results suggest that high-dose radiation may suppress the capacity to synthesize VEGF by disrupting endothelial cell integrity, but antioxidants and radioprotective agents may counteract this effect to some extent. Similarly, our study found that VEGF expression was significantly suppressed in the group receiving single-fraction, high-dose RT. However, this suppression was eliminated in groups that received melatonin and ascorbic acid. Although these studies were conducted in different tissues, the parallel results support the idea that the VEGF-suppressing effect of high-dose RT may develop through a common biological mechanism, and that antioxidant agents may protect the vascular response. Lee et al. investigated radiation-induced cerebrovascular changes by administering a single dose of 10 Gy of gamma radiation to the whole brain of rats [9]. Their study revealed that, compared to the sham-irradiated control group, the rats that received RT exhibited a reduced number of CD31-positive endothelial cells, suppressed endothelial cell proliferation, and increased apoptosis. Furthermore, VEGF, Ang-1, and Tie-2 expression was reported to be significantly decreased, while Ang-2 expression was increased. These findings demonstrate that single-fraction, high-dose, whole-brain RT causes endothelial damage by suppressing the angiogenic balance [9].

Melatonin and ascorbic acid did not increase VEGF expression beyond control levels, indicating that their protective effects are not mediated by the stimulation of angiogenesis but rather by the preservation of vascular integrity. This finding suggests that antioxidant treatment limits radiation-induced vascular and neuronal injury without triggering a compensatory angiogenic response. Supporting this concept, Aras et al. demonstrated that melatonin significantly attenuated oxidative stress and histopathological damage in the cerebral cortex and cerebellum of rats exposed to a single 16 Gy dose of RT. In that study, the protective effects of melatonin were primarily attributed to its antioxidant capacity, as evidenced by improvements in biochemical markers such as superoxide dismutase, catalase, and glutathione peroxidase, rather than changes in angiogenic or vascular growth factors [27]. Although VEGF or other angiogenesis-related parameters were not evaluated in their model, the observed reduction in vascular dilation, edema, and endothelial damage supports the notion that antioxidant-mediated protection can mitigate RT-induced brain injury at the vascular level. Consistent with these findings, the present study demonstrates that melatonin and ascorbic acid preserve VEGF expression at baseline levels following high-dose RT, suggesting a radioprotective mechanism based on endothelial preservation rather than angiogenic activation.

The significant increase in IL-2 expression in the RT group indicates an enhanced radiation-induced inflammatory response. This response is likely due to increased IL-2 levels caused by T cell proliferation. The balanced IL-2 levels in groups treated with melatonin and ascorbic acid support these agents’ immunomodulatory and anti-inflammatory effects. These results are consistent with previous studies showing that melatonin and ascorbic acid inhibit inflammatory cytokines [6, 28].

The study has some limitations. These include the small sample size, single-dose and single-time-point assessment, and lack of support from oxidative stress and hypoxic molecular biomarkers. Additionally, the absence of long-term neurological and functional assessments limits interpretation of the clinical implications of the findings. Another limitation is that the control group did not undergo a sham injection to account for potential injection-related stress. This study was designed as an exploratory preclinical investigation, and no formal post-hoc power analysis was performed. In particular, subtle effect sizes or potential interaction effects might require larger cohorts for more definitive evaluation.

In conclusion, this study demonstrates that melatonin and ascorbic acid reduce histopathological neural damage and vasocongestion in RT-induced brain injury, exhibiting protective effects at vascular and immunological levels. However, combined use was found to not provide a synergistic advantage over single applications. Further research involving different doses, timing, and long-term follow-up is needed to more clearly demonstrate the clinical potential of these agents in reducing radiation neurotoxicity.

## Data Availability

The dataset used and/or analyzed during the current study available from the corresponding author on reasonable request.
